# Optimal Route for Human Umbilical Cord Blood-Derived Mesenchymal Stem Cell Transplantation to Protect Against Neonatal Hyperoxic Lung Injury: Gene Expression Profiles and Histopathology

**DOI:** 10.1371/journal.pone.0135574

**Published:** 2015-08-25

**Authors:** Dong Kyung Sung, Yun Sil Chang, So Yoon Ahn, Se In Sung, Hye Soo Yoo, Soo Jin Choi, Soo Yoon Kim, Won Soon Park

**Affiliations:** 1 Department of Pediatrics, Samsung Medical Center, Sungkyunkwan University School of Medicine, Seoul, Korea; 2 Samsung Biomedical Research Institute, Samsung Medical Center, Sungkyunkwan University School of Medicine, Seoul, Korea; 3 Biomedical Research Institute, MEDIPOST Co., Ltd., Seoul, Korea; Wake Forest Institute for Regenerative Medicine, UNITED STATES

## Abstract

The aim of this study was to determine the optimal route of mesenchymal stem cell (MSC) transplantation. To this end, gene expression profiling was performed to compare the effects of intratracheal (IT) versus intravenous (IV) MSC administration. Furthermore, the therapeutic efficacy of each route to protect against neonatal hyperoxic lung injury was also determined. Newborn Sprague-Dawley rats were exposed to hyperoxia (90% oxygen) from birth for 14 days. Human umbilical cord blood-derived MSCs labeling with PKH26 were transplanted through either the IT (5×10^5^) or IV (2×10^6^) route at postnatal day (P) 5. At P14, lungs were harvested for histological, biochemical and microarray analyses. Hyperoxic conditions induced an increase in the mean linear intercept and mean alveolar volume (MAV), indicative of impaired alveolarization. The number of ED-1 positive cells was significantly decreased by both IT and IV transplantations. However, IT administration of MSCs resulted in a greater decrease in MAV and ED-1 positive cells compared to IV administration. Moreover, the number of TUNEL-positive cells was significantly decreased in the IT group, but not in the IV group. Although the IT group received only one fourth of the number of MSCs that the IV group did, a significantly higher number of donor cell-derived red PKH 26 positivity were recovered in the IT group. Hyperoxic conditions induced the up regulation of genes associated with the inflammatory response, such as macrophage inflammatory protein-1 α, tumor necrosis factor-α and inter leukin-6; genes associated with cell death, such as p53 and caspases; and genes associated with fibrosis, such as connective tissue growth factor. In contrast, hyperoxic conditions induced the dwon-regulation of vascular endothelial growth factor and hepatocyte growth factor. These hyperoxia-induced changes in gene expression were decreased in the IT group, but not in the IV group. Thus, local IT MSC transplantation was more effective than systemic IV MSC administration in protecting against neonatal hyperoxic lung injury.

## Introduction

Bronchopulmonary dysplasia (BPD) is a chronic pulmonary disease that affects very premature infants receiving prolonged ventilator support and oxygen supplementation [1, 2]. Despite recent improvements in neonatal intensive care medicine, BPD still is a major cause of mortality and morbidity among premature infants [3]. Currently, few effective treatments are available to improve BPD prognosis. Therefore, novel therapeutic modalities are urgently needed to improve the outcome of this disease.

Previously we have shown that administration of human umbilical cord blood (UCB)-derived mesenchymal stem cells (MSC) attenuated hyperoxic lung injury in newborn rats [4]. The protective effects of MSC transplantation were also shown to be time-dependent [5], and dose-dependent [6]. Moreover, human UCB-derived MSC transplantation into preterm infants is safe and feasible as demonstrated in a phase I dose escalation clinical trial [7]. Overall, these findings suggest that transplantation of human UCB-derived MSCs could be a novel therapeutic modality for BPD in premature infants.

Determining the optimal route of MSC transplantation is essential in achieving successful clinical translation. Although both local intratracheal (IT) [4] and systemic intravenous (IV) [4] transplantation are feasible, the optimal route for MSC transplantation has not been elucidated yet. We have previously shown that local intratracheal (IT) transplantation of human UCB-derived MSCs is more effective than systemic intraperitoneal (IP) administration in protecting against neonatal hyperoxic lung injury [4]. However, since IP transplantation of MSCs has been shown to be less effective than IV administration in attenuating amidarone-induced lung injury in rats [8], it is important to determine whether local IT MSC transplantation is more effective to systemic IV MSC administration for protection against hyperoxic neonatal lung injury.

Moreover, although the protective effects of MSCs are known to be mediated by paracrine factors rather than a regenerative mechanism [9], the precise paracrine mediators responsible for these effects have not yet been elucidated. In the present study, we sought to determine the optimal route of MSC transplantation. To this end, we compared the therapeutic efficacies of local IT versus systemic IV transplantation of human UCB-derived MSCs to protect against hyperoxia-induced lung injury in newborn rats. We also evaluated whether route-dependent variations in therapeutic efficacy are associated with or mediated by alterations in gene expression profile.

## Materials and Methods

### Animal model

All animal procedures were approved by the Institutional Animal Care and Use Committee of Samsung Biomedical Research Institute, Seoul, Korea. These procedures were also performed in accordance with our institutional guidelines, in addition to the National Institutes of Health Guidelines for Laboratory Animal Care. All animal procedures were performed in an AAALAC-accredited specific pathogen-free facility. Timed pregnant Sprague Dawley rats (Orient Co., Seoul, Korea) spontaneously delivered newborn rat pups as described previously[5, 6]. Newborn SD rats, those reared with their dams in the standard cage, 50 liter Plexiglas chamber, were used in this study. Dam rats could access to water and laboratory chow freely, and were maintained in an alternating 12-hour light/dark cycle with constant room humidity and temperature. We assessed and monitored the condition of rat pups on a weekly basis regularly and twice per day in a daily basis especially for the 14 days after hyperoxia exposure. In this study, we used humane endpoint as the earliest indicator in an animal experiment of pain or distress that could be used to avoid or limit pain and distress by taking actions such as humane euthanasia. For humane endpoint, operationally defined scoring system was approved by IACUC. Total scores of with or more than 5 or score 3 in any single category were arbitrarily defined as humane endpoint. Humane endpoints consist of body weight growth (1: slower growth than normal rats, 2: growth arrest, 3: weight loss), responsiveness (1: delayed but appropriate response, 2: delayed and null response, 3: no response), and appearance (1: rough hair coat, 2: porphyrin staining, 3: sustained abnormal posture or dilated pupil).Throughout the experimental period, no rat pups reached a humane endpoint. Newborn rats were randomly allocated to four experimental groups: normoxia control group (NC, n = 18), hyperoxia control group (HC, n = 18), hyperoxia with IT human UCB-derived MSC transplantation group (HT, n = 18), and hyperoxia with IV human UCB-derived MSC transplantation group (HV, n = 18). Normoxic rats were raised in a room air, whereas hyperoxic rats were raised in hyperoxic chambers containing 90% oxygen from birth until postnatal day (P) 14. IT administration was performed under inhalation anesthesia induced with a mixture of halothane and 2:1 nitrous oxide: oxygen and all efforts were made to minimize suffering. At P14, rat pups were sacrificed under deep pentobarbital anesthesia (60 mg/kg, IP), and lung tissue was harvested for morphometric (n = 7 per group), biochemical (n = 7 per group) and microarray (n = 4 per group) analyses, as described previously [5].

### Preparation of MSCs and transplantation

Human UCB was collected from the umbilical vein after neonatal delivery, and human UCB-derived MSCs were prepared according to the proper manufacturing practices at MEDIPOST Co., Ltd.(Seoul Korea). Mother was provided informed consent. UCB collection and MSC isolation of from UCB were approved by the Institutional Review Board of MEDIPOST Co., LTD. In the present study, we used MSCs from single donor to avoid donor related variation. Cell quality control and quality assurance tests were conducted in accordance with KFDA standards. MSCs were cultivated as previously described [10, 11] and additional details are described in our previous reports [5–7, 12]. Before transplantation human UCB-derived MSCs (fifth passage, single donor) were labeled using a PKH26 Red Fluorescent Cell Membrane Labeling Kit (Sigma-Aldrich, St. Louis, MO, USA) according to the manufacturer protocol. On P5, a single dose of human UCB-derived MSCs was administered either intratracheally (5 × 10^5^ cells) or intravenously (2 × 10^6^ cells) via the right jugular vein. Transplantation timing referred to our previous study. Transplantation timing was based upon our previous study [5].

### Quantification of PKH26-Positive Cells

Cryosections (10 μm thick) were mounted with a VECTASHIELD mounting medium containing DAPI (H-1200, Vector Laboratories, Burlingame, CA, USA). After combining the ×20 objective images of DAPI-stained nuclear signals, the numbers of PKH26 red fluorescence signal were counted manually and averaged per high-power field (HPF) in a single animal. Five fields per section were randomly selected, focused, and counted with the naked eye under a fluorescence microscope (Nikon E600, Nikon, Tokyo, Japan). Two random sections per animal were evaluated in a blinded manner.

### Morphometric Analysis

Paraffin sections (4 μm thick) were stained with hematoxylin and eosin. Two sections were randomly chosen per rat, and three random microscope fields of the distal lung were analyzed for each section. The level of alveolarization was determined by measuring the mean linear intercept (MLI) and mean alveolar volume (MAV) as previously described [4].

### Microarray Analysis and Functional Analysis

Total RNA was isolated using Trizol reagent according to the manufacturer’s instructions (Invitrogen, Waltham, MA, USA). For microarray analysis, RNA was hybridized to Rat Oligo Microarray (44K) chips (Agilent Technology, Palo Alto, CA, USA). Next, the hybridized images were scanned using a DNA microarray scanner (Agilent Technology) and quantified with the Feature Extraction software (Agilent Technology). All data normalization and selection of fold-changed genes were performed using the GeneSpringGX 7.3 software (Agilent Technology). All genes meeting the cut-off criteria were further analyzed by hierarchical clustering and K-means clustering using the GENOWIZ Data Mining Tool (Ocimum Biosolutions, India). For functional analysis, genes were analyzed using DAVID (http://david.abcc.ncifcrf.gov/), Medline (http://www.ncbi.nlm.nih.gov/) and the KEGG (http://www.genome.jp/kegg/) database.

### TUNEL Staining

Apoptotic cells were stained using an ApopTag Fluorescein Apoptosis Detection Kit (Chemicon, Temecula, CA, USA) according to the manufacturer protocol. Slides were mounted in VECTASHIELD mounting medium containing DAPI and visualized by fluorescence microscopy. The number of TUNEL-positive cells was determined in 10 non-overlapping random fields per animal in a blinded manner.

### Quantification of ED-1-positive cells

Immunohistochemical analysis of reactive microglia (ED-1) was performed on deparaffinized 4-μm lung sections. Specimens were placed in a solution containing 0.1% (v/v) Triton X-100 and 0.5%(v/v) BSA in PBS and incubated with primary anti-monocyte/macrophage antibodies (1:100; anti-CD68 ED-1 mouse monoclonal, Chemicon, Millipore, MA, USA). Sections were then stained with fluorescein isothiocyanate (FITC)-conjugated polyclonal rabbit anti-mouse immunoglobulins(1:1000, 1:200) for 1 h at room temperature. VECTASHIELD mounting medium with DAPI (Vector Laboratories) was used to counterstain nuclei.ED-1-positive cells were counted manually and averaged per HPF in a single animal. Two random sections per animal were evaluated in a blinded manner.

### ELISA assay

Frozen lung samples were homogenized in lysis buffer, and lysates were clarified by centrifugation at 13,000 *g* for 20 min at 4°C to remove cellular debris. The protein contents of the supernatants were quantitated by the Bradford method, using bovine serum albumin as the standard. Lung macrophage inflammatory protein-1 α (MIP-1α), tumor necrosis factor- α (TNF-α) and interleukin-6 (IL-6) levels were measured using the Milliplex MAP ELISA Kit according to the manufacturer’s protocol (Millipore, Billerica, MA, USA). Vascular endothelial growth factor (VEGF) levels were measured using the R & D Rat VEGF Quantikine ELISA kit according to the manufacturer’s protocol (R&D Systems, Minneapolis, MN, USA).

### RT-PCR

The total RNA concentration was measured at 260 nm using a nanodrop spectrophotometer (Nanodrop Wilmington, DE, USA). One microgram of RNA was reverse transcribed into cDNA with a Protoscript II RT-PCR kit (New England Biolabs, Ipswich, MA, USA). PCR primers were synthesized by Bioneer Inc. (Daejeon, Korea). The primer sequences used were as follows: rat hepatocyte growth factor (HGF) (sense-ACCCTGGTGTTTCACAAGCA-, antisense-AGGGGTGTCAGGGTCAAGAG-), rat p53 (sense-AGCGTGGTGGTACCGTATGA-, antisense-TCAGCTCTCGGAACATCTCG-), rat caspase3 (sense- GGTATTGAGACAGACAGTGG-, antisense- CATGGGATCTGTTTCTTTGC-), rat connective tissue growth factor (CTGF) (sence-CAAGCTGCCCGGGAAAT-, antisense-CGGTCCTTGGGCTCATCA-), and rat GAPDH (sense- GGCCAAAGGGTCATCATCT-, antisense-GTGATGGCATGGACTGTGGT-). PCR products were resolved by electrophoresis on a 1.2% agarose gel, visualized by ethidium bromide and scanned with a Gel Doc 2000 analyzer (Bio-Rad Laboratories, Inc. Hercules, CA, USA). The expression level of each gene was determined semi-quantitatively by densitometric analysis using the Quantity One software (Bio-Rad Laboratories, Inc.). Relative expression levels were estimated by normalizing to the density of the appropriate rat GAPDH band.

### Statistical analysis

Data are expressed as means ± SEM. Survival curves were compared using Kaplan-Meier analysis followed by a log rank test. Microarray data were analyzed using one-way analysis of variance (ANOVA) with a Benjamini–Hochberg correction for multiple comparisons (GeneSpring, Agilent Technologies, Santa Clara, CA, USA). Normality test was performed for all the parameters. One-way ANOVA followed by Tukey’s multiple comparison tests were used for group comparisons of the parameters that were normally distributed. For non-parametric values, such as cell counts that were not normally distributed, the Kruskal-Wallis test was performed for group-comparisons. P-values < 0.05 were considered significant. SPSS software version 17.0 (SPSS Institute, Chicago, IL, USA) was used for all analyses.

## Results

### Survival Rate and Body Weight Gain

**Fig 1:**
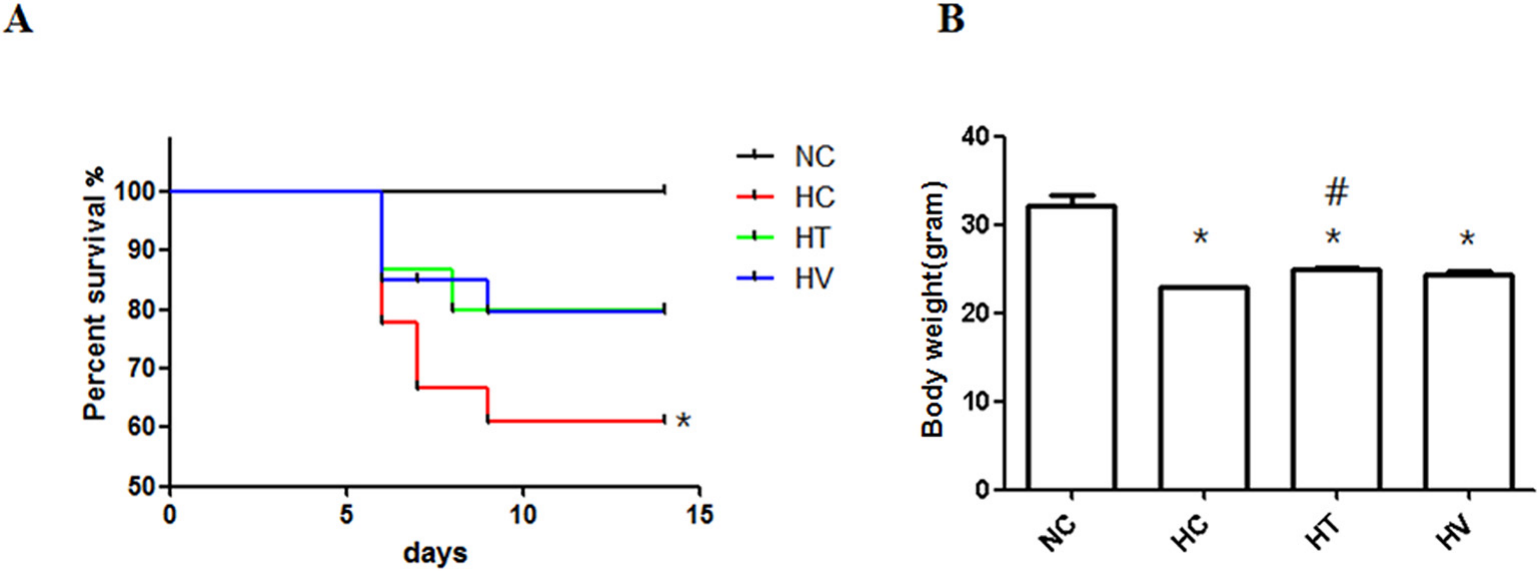
Survival rate and weight growth. (A) Kaplan-Meier survival curve and (B) body weights at postnatal day (P) 14. Normoxia control (NC), hyperoxia control (HC), hyperoxia-exposed rats with intratracheal transplantation of MSCs (HT) and hyperoxia-exposed rats with intravenous transplantation of MSCs (HV) groups, respectively.* *P* < 0.05 vs. NC; ^#^ *P* < 0.05 vs. HC (n = 18 per group).

Exposure to oxygen (HC group) significantly reduced the survival rate (*P* < 0.05 vs. NC) by the end of experiment (P14), compared to the 100% survival rate of the NC group. On the contrary, the survival rates of the HT and HV groups were not different when compared to the NC group. There was no difference in the survival rate between the HT and HV groups (Fig 1A).The decrease in body weight gain observed in the HC group was significantly improved in the HT group (*P* < 0.05 vs. HC), but not in the HV group (Fig 1B).

### Donor cell localization in P14 rat lungs

**Fig 2:**
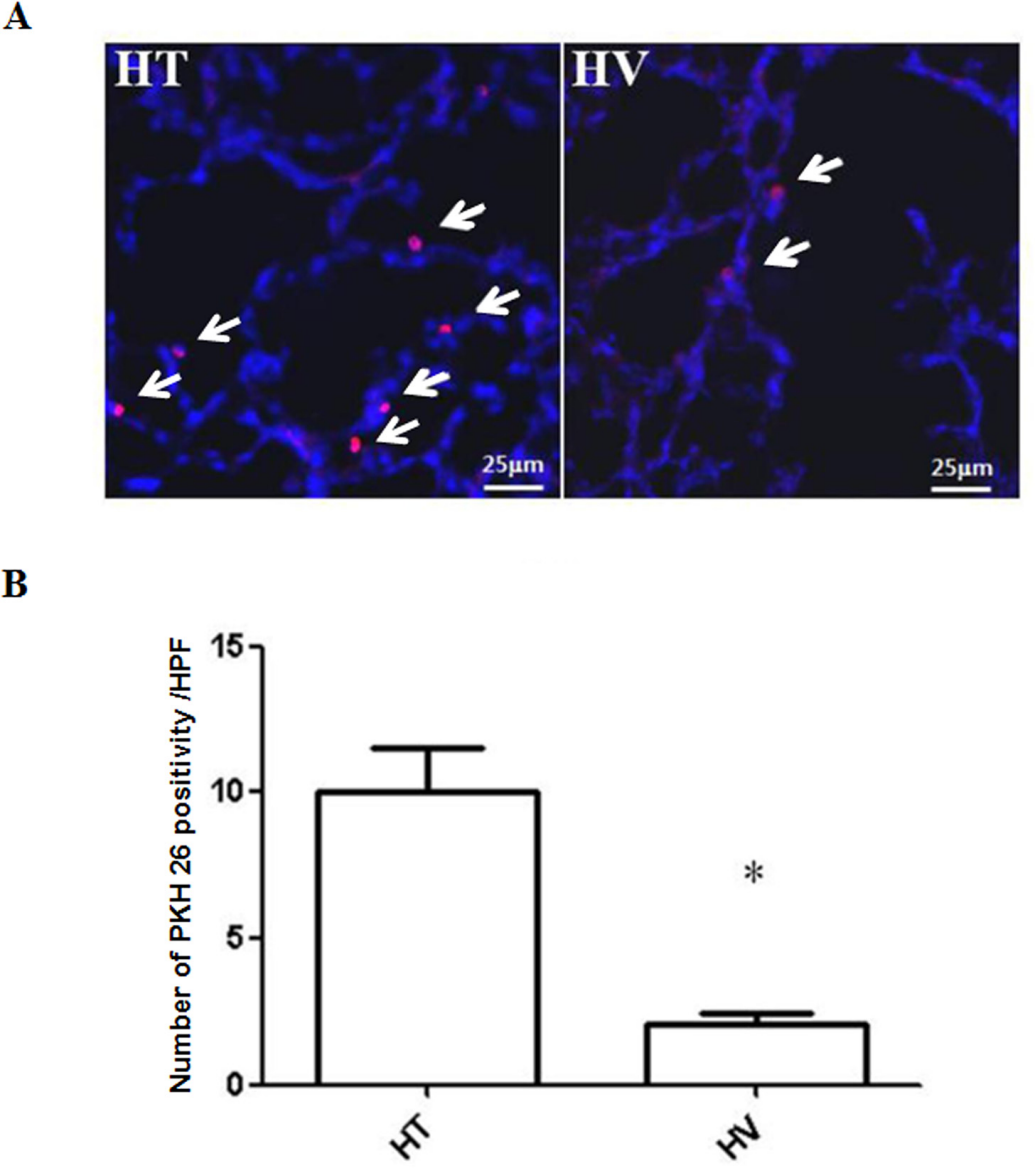
Donor cell derived PKH 26 positivity in the lungs of P14 rats. Top panels: Fluorescence microscope images of donor MSC-derived PKH26 positivity (arrows, red) localized in the lungs of P14 newborn rats. Nuclei were counterstained with DAPI (blue) (scale bars: 25 μm, x400). More number of red fluorescent positivity was detected in the HT group than in the HVgroup. Bottom panels: Numbers of PKH26-positivity in the lung tissue per high-power field. * *P <* 0.05 vs. NC (n = 7 per group).

The deposition of donor cell-derived PKH26-positivity (red fluorescence) was detected only in the MSC transplantation groups (HT and HV) and not in the NC or HC groups (Fig 2A). The number of PKH26-positivity derived from donor cells identified per lung field was significantly higher in the HT group than in the HV group (Fig 2B).

### Histology and morphometrics of P14 rat lungs

**Fig 3:**
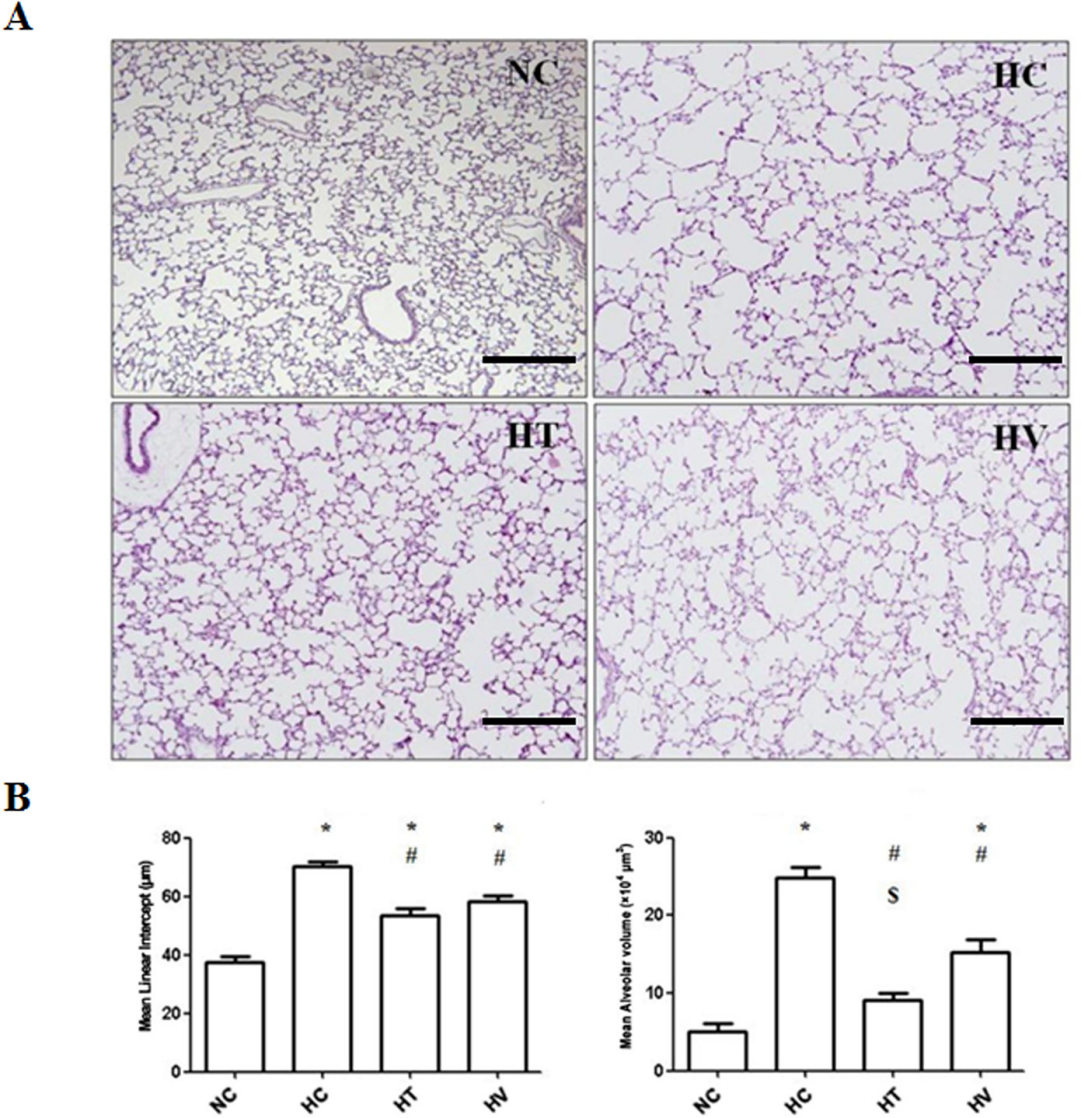
Histological and morphometric analysis of lung tissue from P14 rats. Hematoxylin and eosin-stained lung sections from representative animals (scale bar = 200 μm, × 100). The NC group shows normal alveolar development. The HC group shows disrupted alveolar development, with larger and simpler alveoli. The HT and HV groups both show attenuated lung morphometric changes upon stem cell transplantation. The degrees of alveolarization, as measured by the mean linear intercept (MLI) and mean alveolar volume (MAV), of P14 rat lungs are shown. MLI and MAV in the HT and HV groups are significantly lower than in the HC group. MAV in the HT group is significantly lower than in the HC and HV group. * *P* < 0.05 vs. NC; ^#^ *P* < 0.05 vs. HC; ^$^ *P* < 0.05 vs. HV (n = 7 per group).

Representative light microscope photomicrographs showing the histopathological differences between the experimental groups are shown in Fig 3A. While small and uniform alveoli were observed in the NC group, the alveoli in the HC group were fewer and larger. The HC group also exhibited focal airspace enlargement and heterogeneous alveolar sizes, which are indicative of an impaired alveolarization. These hyperoxia-induced morphological changes and the impaired alveolar growth were attenuated after MSC transplantation, particularly in the HT group compared to the HV group. Morphometric analysis revealed that the MLI and MAV, which represent the mean size and volume of the alveoli respectively, were significantly higher in the HC group than in the NC group (Fig 3B). These hyperoxia-induced morphometric abnormalities were significantly attenuated in the HT and HV groups, with a greater attenuation of the alveolar volume in the HT group than in the HV group.

### Gene expression profiling after hyperoxia treatment and MSC transplantation

To identify alterations in gene expression resulting from the hyperoxia treatment and MSC transplantation, microarray analyses were performed on lung RNA from each experimental group. A hierarchical clustering dendrogram representing the relative gene expression in each group was generated using GeneSpring GX 7.3 Software (Agilent Technology) (S1A Fig). Changes in gene expression were considered significant if greater than 2 fold with a *P* value less than 0.05.

In the HC group, the expression of 15,728 genes underwent significant changes (8,263 upregulated and 7,465 down-regulated genes) compared to the NC group according to the GeneSpringGX change algorithm criteria. To identify groups of genes with similar expression patterns, the 15,728 genes were clustered into eight different groups using the K-means clustering approach (S1B Fig).

Of the 1,231 genes in clusters 3 and 4, 183 were significantly upregulated in the HC group compared to the NC group. Moreover, these 183 genes were significantly down regulated in the HT group, but not in the HV group (S2A Fig). Thus, this group of 183 genes was termed the “HC high/HT low” group. Of the 512 genes in cluster 5, 142 were significantly down regulated in the HC group compared to the NC group. These 142 genes were also significantly upregulated in the HT group, but not in the HV group (S2B Fig). Thus, this group of 142 genes was termed the “HC low/HT high” group.

**Fig 4:**
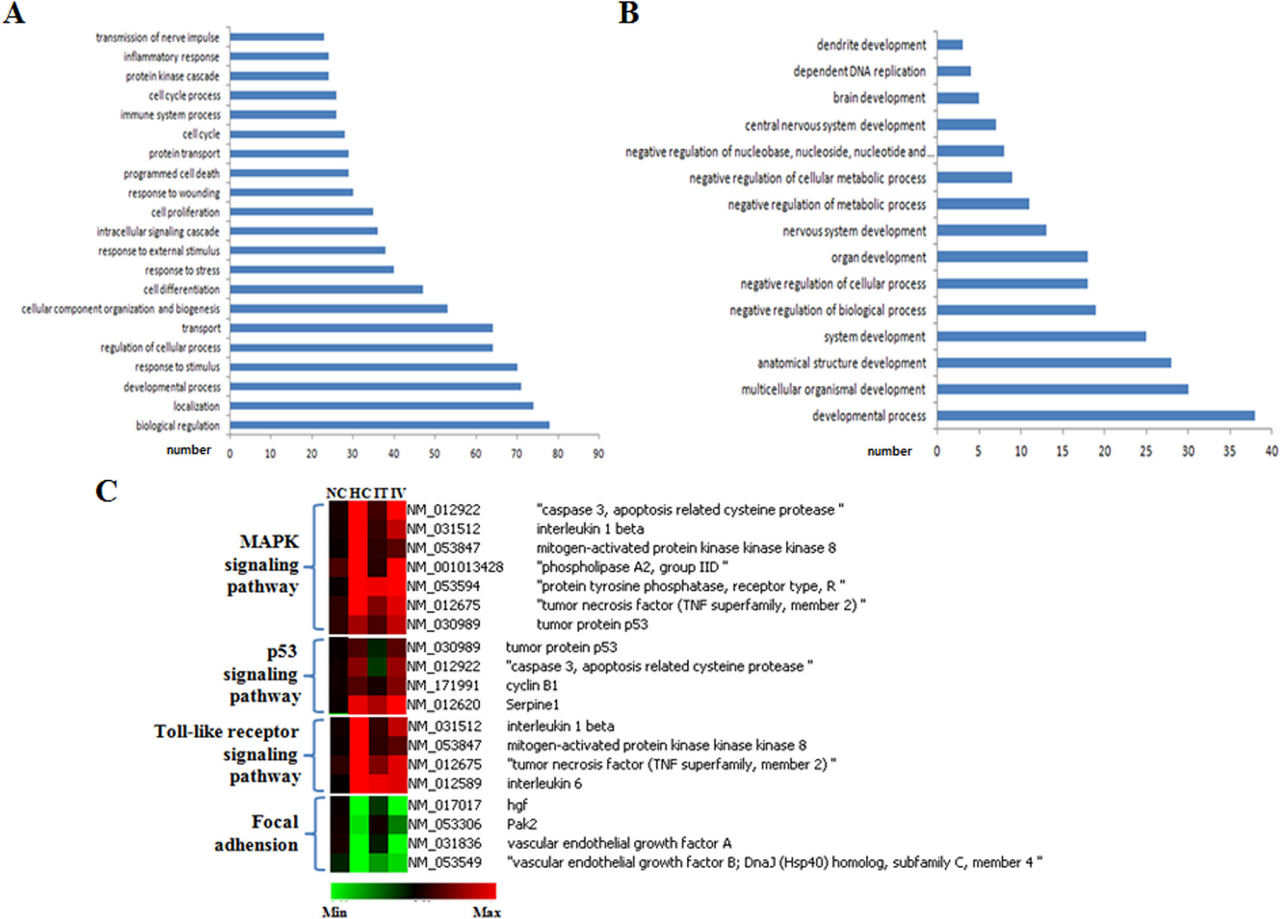
Functional annotation and KEGG molecular pathway analysis. (A) Enriched functional categories in the HC high/HT low group (B) Enriched functional categories in the HC low/HT high group. (C) KEGG pathway heat map of the differentially expressed genes. The HC high/HT low group was linked to MAPK, p53, and Toll-like receptor signaling pathways. The HC low/HT high group was linked to focal adhesion and renal cell carcinoma.

The HC high/HT low and HC low/HT high groups were analyzed for functional annotation and KEGG molecular pathway enrichment. Gene ontology analysis of the 183 differentially expressed genes in the HC high/HT low group revealed that they were related to developmental processes, immune response and cell death (Fig 4A). KEGG molecular pathways enriched in this group included MAPK, p53, and Toll-like receptor signaling pathways (Fig 4C, Table 1).

**Table 1:** KEGG pathway analysis of up regulated genes in the HC high/HT low group.

Pathway description	Number of genes	p-value	Genes
**MAPK signaling pathway**	7	0.008382503	caspase 3, interleukin 1 beta, mitogen-activated protein kinase kinasekinase 8,phospholipase A2, protein tyrosine phosphatase, tumor necrosis factor-alpha, p53
**Alzheimer's disease**	6	0.010068981	tumor necrosis factor-alpha, interleukin 1 beta, nitric oxide synthase 1, caspase 3, NADH dehydrogenase flavoprotein 1, NADH dehydrogenase Fe-s protein 7
**Amyotrophic lateral sclerosis (ALS)**	4	0.007249765	caspase 3, tumor necrosis factor-alpha, p53, nitric oxide synthase 1
**NOD-like receptor signaling pathway**	4	0.008317088	chemokine(C-X-C motif) ligand 1, interleukin 1 beta, interleukin 6, tumor necrosis factor-alpha
**p53 signaling pathway**	4	0.00987751	caspase 3, cyclin B1, serine(or cysteine) peptidase inhibitor, p53
**Hematopoietic cell lineage**	4	0.015530477	interleukin 1 beta, interleukin 6, transferrin receptor, tumor necrosis factor-alpha
**Apoptosis**	4	0.019519107	caspase 3, interleukin 1 beta, tumor necrosis factor-alpha, p53
**Toll-like receptor signaling pathway**	4	0.022685012	interleukin 1 beta, interleukin 6, tumor necrosis factor-alpha, mitogen-activated protein kinase kinasekinase 8
**Prion diseases**	3	0.023252997	interleukin 1 beta, interleukin 6, prion protein
**Graft-versus-host disease**	3	0.046566578	interleukin 1 beta, interleukin 6, tumor necrosis factor-alpha

The HC high/HT low group was analyzed for functional annotation by KEGG pathway mapping.

Gene ontology analysis of the 142 differentially expressed genes in the HC low/HT high group revealed that they were related to developmental and metabolic processes (Fig 4B). KEGG molecular pathways enriched in this group included focal adhesion and renal cell carcinoma (Fig 4C, Table 2).

**Table 2:** KEGG pathway analysis of down regulated genes in the HC low/HT high group.

Pathway description	Number of genes	p-value	Genes
**Renal cell carcinoma**	4	0.002041679	VEGF A, VEGF B, HGF, p21 protein
**Focal adhesion**	4	0.035000541	VEGF A, VEGF B, HGF, p21 protein

The HC low/HT high group was analyzed for functional annotation by KEGG pathway mapping.

### TUNEL and ED-1 staining

**Fig 5:**
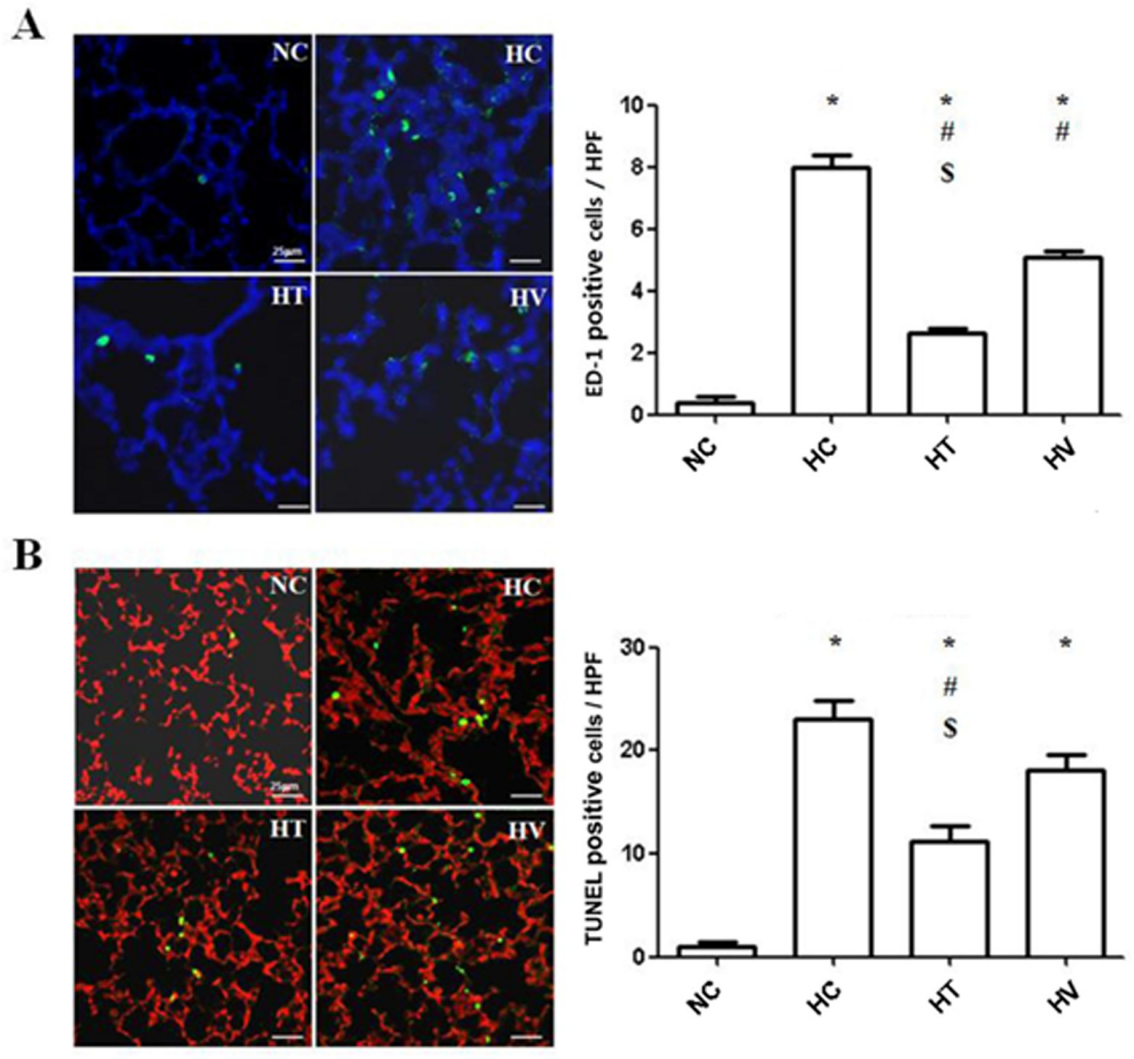
Analysis of ED-1-positive cells and apoptotic cells. (A) Both HC and HV groups exhibited significantly more ED-1-positive cells than the HT group. ED-1 positive cells were labeled with FITC (*green*), and nuclei were labeled with DAPI (blue) (scale bars: 25 μm, × 400). (B) Apoptotic cells in the lungs of neonatal rats exposed to hyperoxic (90% oxygen) or normoxic conditions were identified by *in situ* TUNEL. Apoptotic cells were labeled with FITC (*green*), and nuclei were labeled with PI (red) (scale bars: 25 μm, × 400). The lungs of rat lungs exposed to hyperoxic conditions exhibited increased numbers of apoptotic cells compared to control rats. Less TUNEL-positive cells were detected in the HT group than in the HT and HV groups.* *P* < 0.05 vs. NC; ^#^ *P* < 0.05 vs. HC; ^$^ *P* < 0.05 vs. HV. (n = 7 per group)

The numbers of ED-1-positive and TUNEL-positive cells per high power field were significantly higher in the HC group than in the NC group (Fig 5). This hyperoxia-induced increase in the number of ED-1 positive cells was significantly attenuated in both the HT and HV groups, with more attenuation in the HT group (Fig 5A). Moreover, the increase in the number of TUNEL-positive cells observed in the HC group was significantly attenuated only in the HT group and not in the HV group (Fig 5B).

### Validation of microarray data by histological and biochemical evaluation

To validate the microarray results, the expression of eight genes with important roles in inflammation, angiogenesis, fibrogenesis and cell death was examined using either RT-PCR or ELISA in each experimental group.

**Fig 6:**
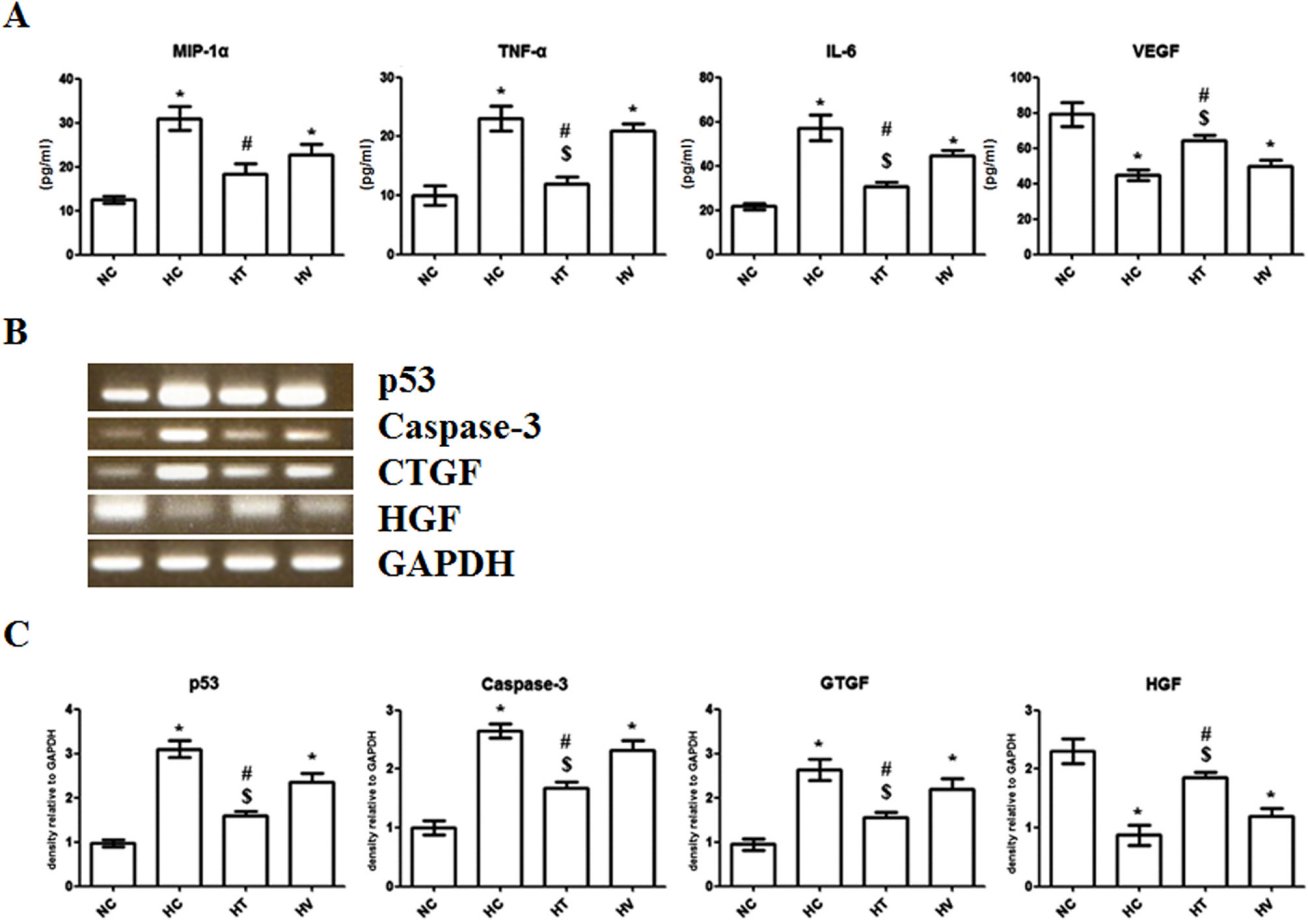
Validation of protein levels and mRNA expression of inflammatory cytokines and other genes identified by microarray analysis. (A) Protein levels of the inflammatory cytokines MIP-1α, TNF-α and IL-6, and VEGF in lung homogenates were quantified by ELISA. Cytokine levels were significantly decreased in the HT group compared to the HC group. The level of VEGF was significantly increased in the HT group compared to the HC and HV groups. (B) Representative PCR gels for p53, caspase-3, CTGF, and HGF genes. (C) The relative expression of the apoptotic genes of P53 and caspase-3, CTGF, and HGF in lung homogenates analyzed by RT-PCR. Relative gene expression of P53, caspase-3, and CTGF was lower in the HT group than in the HC and HV groups. The relative expression of HGF gene was higher in the HT group than in the HC and HV groups. * *P* < 0.05 vs. NC; ^#^, *P* < 0.05 vs. HC; ^$^ *P* < 0.05 vs. HV (n = 7 per group).

In concordance with the microarray data of the 183 genes in the HC high/HT low group, RT-PCR and ELISA analyses confirmed the involvement of this group in the inflammatory response (Toll-like receptor pathways), cell death (apoptosis pathways) and fibrosis, which had been predicted by KEGG and DAVID analyses. Specifically, the protein levels of MIP-1α, TNF- α and IL-6 measured by ELISA were significantly increased in the HC group compared to the NC group. Moreover, this increase was only significantly attenuated in the HT group and not in the HV group (Fig 6A). RT-PCR data indicated that the relative expression of p53, caspase 3 and CTGF was higher in the HC and HV group compared to the NC group and lower in the HT group than in the HC and HV groups (Fig 6B and 6C).

In concordance with the microarray data of the 142 genes in the HC low/HT high group, which described an involvement in angiogenesis and lung alveolus development according to KEGG and DAVID analyses (Fig 4B), the protein levels of VEGF measured by ELISA were significantly reduced in the HC group compared to the NC group. Moreover, this reduction was only significantly attenuated in the HT group and not in the HV group (Fig 6A). RT-PCR data indicated that the relative expression of the HGF gene was lower in the HC and HV than in the NC group and higher in the HT group than in the HC and HV groups (Fig 6B and 6C).

## Discussion

Exposure of newborn rat lung to hyperoxia throughout the neonatal period closely mimics the histopathology observed in human infants with BPD including impaired alveolarization and vascular growth [13–15]. Moreover, as the saccular stage of the newborn rat pup lung at birth corresponds to the lung developmental stage of premature infants at 25 week of gestation, newborn rat pups ideally contain an element of lung immaturity [14, 15]. As the extent of lung injury is proportional to the concentrations of oxygen, rat pups are exposed to high oxygen concentrations, usually above 90%, to study a severe hyperoxic lung injury [4–6, 16–18]. However, more subtle but definite hyperoxic lung injuries are also observed following an exposure to 40% to 60% of oxygen, which is still moderately high but closer to the oxygen levels used clinically in preterm infants [13, 15]. Although the duration of hyperoxic exposure starting from the saccular stage could vary, in most studies the rat pups are kept in hyperoxia for up to 14 days after birth, which is equivalent to the alveolar stage and early infancy in the human [13–15]. Hence the newborn rat pup exposed to hyperoxia is a useful animal model for studying BPD.

Determining the optimal route of MSC transplantation is essential for successful clinical translation of stem cell therapy for BPD. When compared with the more invasive local IT approach for MSC administration, the minimally invasive and practical systemic IV approach exhibits distinct therapeutic advantages, especially considering that many patients with BPD are clinically unstable preterm neonates. However, some animal studies have suggested that the IV route is not optimal for treating local pulmonary lesions because transplanted cells may be trafficked not only in the pulmonary capillaries, but also in other organs such as the liver, spleen and kidneys [19]. In our previous study [4] and in the present study, we found that significantly higher number of donor cell-derived PKH 26 positivity was correctly localized in the IT administration group compared to the IV and the IP transplantation groups. This finding is striking, considering that four-fold higher doses of MSCs were given systemically by IV than locally by IT delivery.

This observation indicates that invasive local IT route may be a more efficient delivery route than the systemic IV route, for the MSC transplantation.

Furthermore, while both IV and IT transplantation of MSCs significantly attenuated many symptoms of hyperoxia-induced lung injury, including impaired alveolarization, inflammatory response, and increased apoptosis; the present study found that the therapeutic efficacy of IT-transplanted MSCs is better than that of IV-administered MSCs. Clinically preterm infants indicated for MSC transplantation are already intubated for ventilator support. In a phase I clinical trial of MSCs for BPD, we demonstrated that IT transplantation in preterm infants (with a mean gestational age of 25 weeks) is safe and feasible, without any adverse outcomes [7]. Taken together, these findings suggest that local IT rather than systemic IV transplantation of human UCB-derived MSCs might be the optimal delivery route for future clinical applications against BPD.

In the present study, differences in MSC accumulation in the lungs between IT and IV groups was assessed by quantification of the red fluorescence positivity because red fluorescent materials originate from donor MSCs not from host cells. However, the possible labeling of donor cell fragments or vesicles by PKH26 staining cannot be excluded in this experiment [20]; thus, PKH26 labeling may not be an ideal method for donor cell tracing. Further studies are needed to clarify this using more reliable donor cell tracing methods.

Despite the fact that IT rather than IV transplantation of MSC at P5 showed a better attenuation of hypeoxic lung injuries in the present study, there was no significant difference in the survival rates. In our previous studies, a higher survival rate was observed when IT MSCs were transplanted at P3 [5], but not at P5 [4, 17]. However, hyperoxic lung injuries were significantly attenuated with IT MSC transplantation not only at P3 but also at P5 [4, 5, 17]. The best indicator for the therapeutic efficacy of a death threatening condition must be survival. Therefore, our discrepant data of significant attenuation of hyperoxic lung injuries, but failure to significantly improve survival, with MSCs transplantation are difficult to explain. Further studies would be necessary to understand why the differences found in histopathology and biochemical disease burden did not lead to differences in survival in this experiment.

The mechanism by which local IT rather than systemic IV MSC transplantation better protects neonatal lung tissue against hyperoxia is of particular interest. Inflammatory processes mediated by cells such as neutrophils and macrophages [14], as well as pro-inflammatory cytokines [21], are known to play a key role in hyperoxia-induced lung injury. These result in impaired alveolarization and angiogenesis, increased apoptosis and fibrosis [16]. The cDNA microarray analysis performed in the present study showed that hyperoxia induces the up regulation of genes involved in inflammation, programmed cell death, and fibrosis. Importantly, the same genes were only significantly down regulated with IT but not with IV MSC transplantation. Furthermore, histological studies revealed that the IT group exhibited fewer ED-1-positive and TUNEL-positive cells than the IV group. Moreover, ELISA showed that the IT group exhibited lower levels of inflammatory cytokines such as MIP-1α, TNF-α and IL-6, in addition to lower levels of cell death-associated proteins such as p53 and caspase-3. Furthermore, lower expression of the fibrosis-associated CTGF gene was assessed by RT-PCR in the IT group than the IV group. Overall, these findings suggest that the ability of IT MSC transplantation to better protect against hyperoxia-induced lung injury is mediated by higher delivery efficiency of this approach, which results in stronger anti-inflammatory effects compared to IV MSC administration.

Although we have demonstrated in our previous study [4] and in the present study that the protective effects of transplanted MSCs against BPD appear to be primarily mediated by their paracrine anti-inflammatory effects rather than their regenerative capabilities, the key trophic factors responsible for these paracrine mechanisms remain to be elucidated. Previously, we demonstrated that hyperoxia-induced decreases in VEGF and HGF levels were significantly improved with human UCB-derived MSC transplantation [13]. Moreover, VEGF knockdown in transplanted MSCs abolishes their protective effects against hyperoxic lung injury. When VEGF knockdown MSCs were unable to attenuate the impaired alveolarization and angiogenesis, the increased TUNEL-positive and ED-1 positive cells, and the up-regulation of pro-inflammatory cytokines resulting from hyperoxic conditions. In the present study, hyperoxia induced the down-regulation of growth factors such as VEGF and HGF, as measured by both cDNA microarray and RT-PCR. Interestingly, these growth factors were only significantly upregulated in the IT MSC transplantation group, but not in the IV group. Our data suggest that the better therapeutic efficacy of IT MSC transplantation compared to IV MSC transplantation might be due to the improved delivery efficiency with the former approach. Moreover, IT rather than IV transplantation appears to be associated with better paracrine potency for the production of trophic factors such as VEGF and HGF. These factors are important because they modify the host lung tissue microenvironment. Taken together, these findings suggest that VEGF and HGF may be the key paracrine factors that mediate protection against hyperoxic lung injury, at least in newborn rats.. There are no doubt that many other factors besides VEGF and HGF play critical roles in the protection of hyperoxic lung injury. Further studies are needed to clarify this.

Another important issue for clinical translation is to determine the optimal dose of MSCs for delivery by either local IT or systemic IV approach. In the present study, four times more MSCs were administered via the systemic IV route than via the local IT route. These doses were based on results from our previous study, which showed less engraftment and reduced therapeutic efficacy against neonatal hyperoxic lung injury with systemic IP transplantation compared to local IT administration, even when four-fold higher doses of MSCs were used in the former approach [4]. The present study shows that better therapeutic efficacy along with more visualization donor cell-derived red fluorescent PKH 26 positivity was achieved with local IT rather than systemic IV MSC transplantation, although both routes were effective. Further studies will be necessary to determine the optimal dose of MSCs to be transplanted via each route for maximal protection against BPD.

In summary, although both local IT and systemic IV delivery routes were effective, the local IT delivery route was more effective than the systemic IV infusion route with respect to delivery efficiency and therapeutic efficacy against neonatal hyperoxic lung injury. Specifically, the local IT delivery route provided better protection against hyperoxia-induced effects, such as impaired alveolarization, increased numbers of TUNEL-positive cells and increased numbers of ED-1-positive cells. Hyperoxia also induced the upregulation of genes associated with inflammation, cell death, and fibrosis, and the dwon-regulation of genes involved in angiogenesis, including VEGF and HGF. These effects were significantly improved in the IT transplantation group but not in the IV group. Our data suggest that local IT rather than systemic IV MSC transplantation is the optimal delivery route for treating premature infants with BPD.

## Supporting Information

S1 Fig Hierarchical clustering and cluster analysis by generating a self-organizing map. (TIF)

S2 Fig Heat map of upregulated and down-regulated genes. (A) 183 out of 1,231 genes were significantly upregulated in the HC group compared to the NC group and down- regulated in the HT group, but not in the HV group. (B) 142 out of 512 genes were significantly down-regulated in the HC group compared to the NC group and upregulated in the HT group, but not in the HV group.
(TIF)
