# Supplementary figures and images for: Optimal Route for Human Umbilical Cord Blood-Derived Mesenchymal Stem Cell Transplantation to Protect Against Neonatal Hyperoxic Lung Injury: Gene Expression Profiles and Histopathology
Source: PLoS One. 2015 Aug 25;10(8):e0135574. doi: 10.1371/journal.pone.0135574 (PMC4549285; doi:10.1371/journal.pone.0135574)

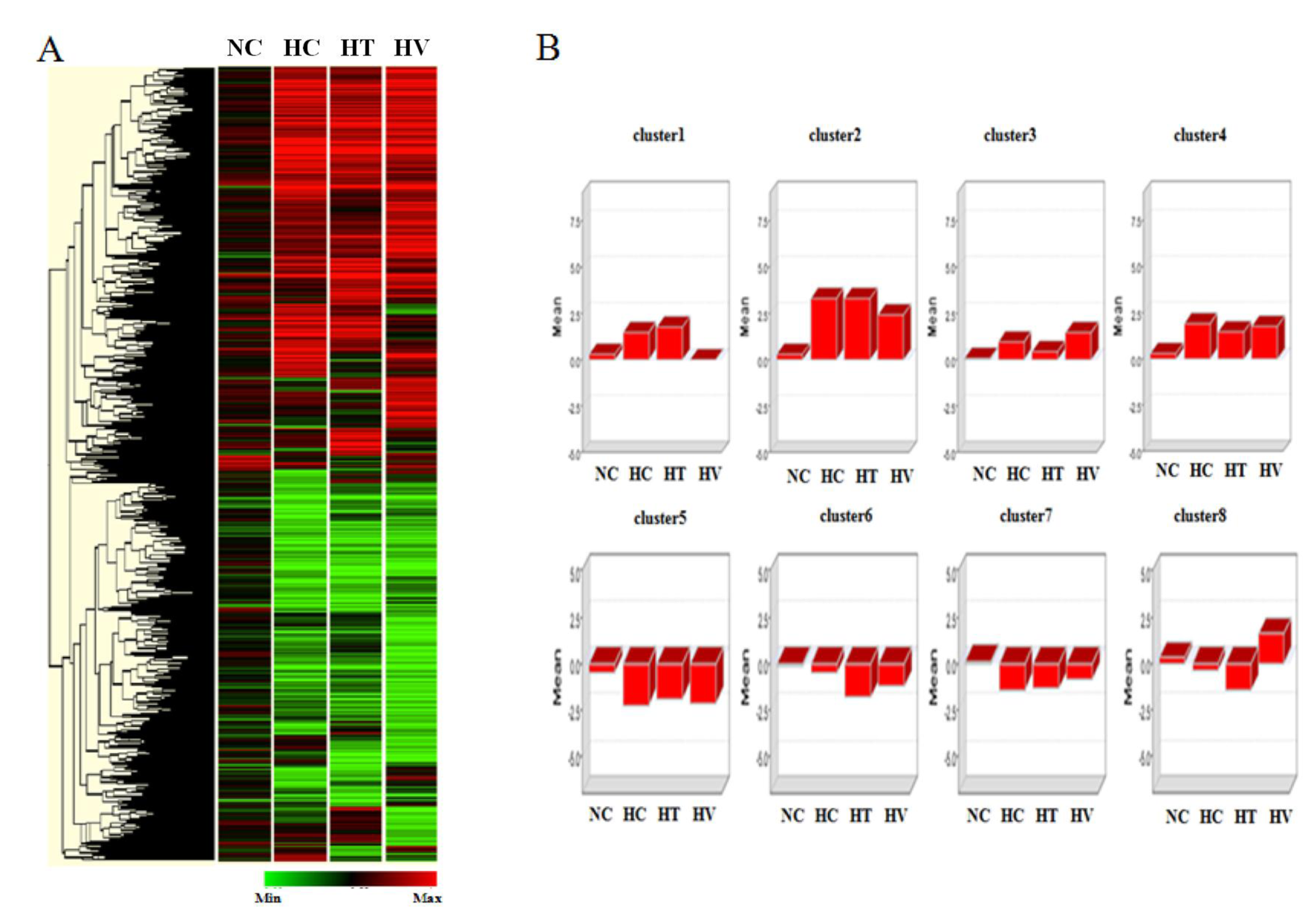

Supplement: S1 Fig — (TIF) [file pone.0135574.s001.tif]

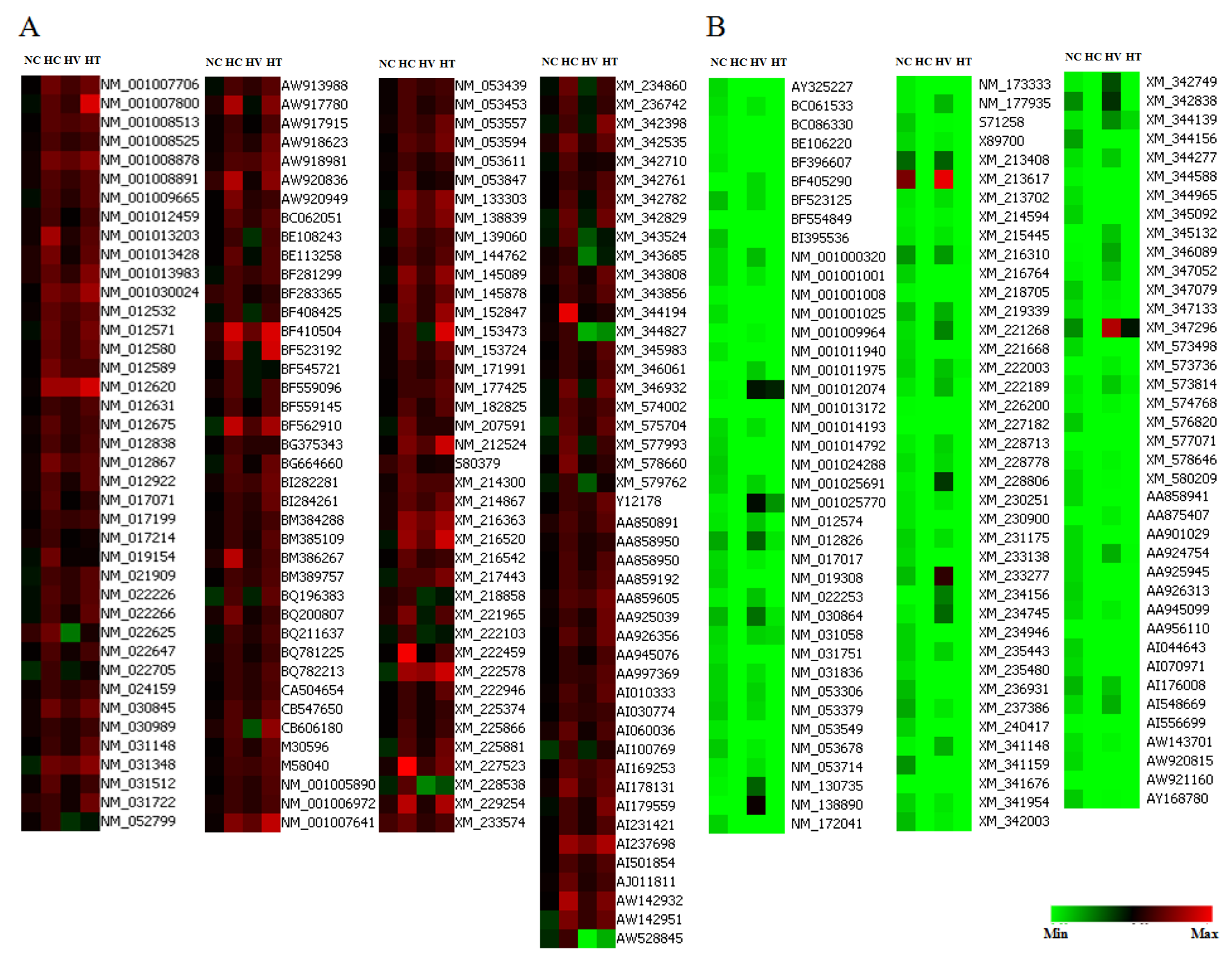

Supplement: S2 Fig — (A) 183 out of 1,231 genes were significantly upregulated in the HC group compared to the NC group and down- regulated in the HT group, but not in the HV group. (B) 142 out of 512 genes were significantly down-regulated in the HC group compared to the NC group and upregulated in the HT group, but not in the HV group. (TIF) [file pone.0135574.s002.tif]
